# The NOP14 nucleolar protein suppresses the function and stemness of melanoma stem-like cells through Wnt/beta-catenin signaling inactivation

**DOI:** 10.1080/21655979.2022.2050491

**Published:** 2022-03-12

**Authors:** Jingrong Li, Ruihua Fang, Jiang Wu, Yuan Si, Jingzhu Bai, Qi Wang

**Affiliations:** a Department of Dermatology, Guangzhou First People’s Hospital, Guangzhou, Guangdong Province, China; b Department of Dermatology, Nanfang Hospital, Southern Medical University, Guangzhou, Guangdong Province, China

**Keywords:** CSCs in melanoma, NOP14, stemness, wnt/beta-catenin signaling

## Abstract

Cancer stem cells (CSCs) are closely related to tumor occurrence, development, metastasis, drug resistance, and recurrence. The role of CSCs in melanoma is poorly understood. Our previous studies suggested that the NOP14 nucleolar protein (NOP14) is involved in melanoma pathogenesis regulation. Importantly, NOP14 overexpression inhibits the Wnt/beta (β)-catenin signaling pathway, an important mechanism regulating CSCs stemness. Therefore, in this study, we aimed to explore the role of NOP14 in the stemness and function of CSCs in melanoma *in vitro*. CD133, a stem cell marker, was used to identify melanoma stem-like cells (SLCs). NOP14 overexpression subsequently decreased the proportion of CD133^+^ SLCs, impaired the colony-forming capabilities, and downregulated the expression of Nanog, SOX2, and OCT4 stem cell markers in A375 and A875 cells, suggesting that NOP14 suppresses the stemness of melanoma SLCs. NOP14 overexpression suppressed the migration, invasion, and angiogenesis-inducing ability of A375-SLCs and A875-SLCs. NOP14 overexpression also inactivated Wnt/β-catenin signaling in melanoma CD133^+^ SLCs. The Wnt signaling activator BML-284 alleviated the effect of NOP14 overexpression on the stemness and function of melanoma CSCs. In conclusion, NOP14 suppresses the stemness and function of melanoma SLCs by inactivating Wnt/β-catenin signaling. Thus, NOP14 is a novel target for CSC treatment in melanoma.

**Abbreviations**: CSCs, cancer stem cells; SLCs, stem-like cells; NOP14, NOP14 nucleolar protein; SCID, severe combined immunodeficiency; β-catenin, beta-catenin; lv-NOP14, lentivirals expressing NOP14; PBS, phosphate buffer saline; HUVECs, human umbilical vein endothelial cells.

## Introduction

Melanoma is a common dermatological tumor characterized by high malignancy, rapid progression, easy metastasis, recurrence, and poor prognosis [1–4]. Radiotherapy and chemotherapy cannot effectively treat malignant melanomas. Diagnostic techniques, surgical strategies, adjuvant chemotherapy, and gene therapy continue to advance and develop, but the prognosis of malignant melanoma has not significantly improved [5]. Studies have shown that the pathogenesis of melanoma is complicated and that melanoma cancer stem cells (CSCs) potentially cause invasion, metastasis, drug resistance, and recurrence [6–8].

CSCs are closely related to tumor occurrence, development, metastasis, drug resistance, and recurrence [9,10] and comprise a small number of cell populations. CSCs function through self-protection mechanisms, such as DNA damage repair, apoptosis pathway inhibition, and the upregulation of drug-resistant proteins [11]. Additionally, human melanoma cells can generate melanoma spheroid cells during culturing [12]. Melanoma cells derived from spheroid cells showed tumorigenicity when injected subcutaneously into severely combined immunodeficiency (SCID) mice. Melanoma spheroid cells cultured from melanoma specimens or cell lines present self-renewal abilities, pluripotency, and tumorigenicity [13]. These studies demonstrate the feasibility of melanoma CSC research. Several signaling pathways are involved in regulating the stemness of melanoma CSCs, including wingless-type MMTV integration site family/beta-catenin (Wnt/β-catenin), Notch, and hedgehog [14–16]. Melanoma CSCs remain poorly understood with no effective strategies to inhibit CSC maintenance and self-renewal. Thus, a deeper understanding of the biological characteristics of CSCs in melanoma is required to elucidate the molecular mechanisms, identify the key regulatory molecules, and provide novel solutions and a theoretical basis for targeted melanoma CSC therapy.

Our previous investigations reported significantly that NOP14 nucleolar protein (NOP14) expression level decreased in melanoma tissues, compared to that in tissues surrounding the melanoma or nevus, and that NOP14 expression is significantly correlated with melanoma tissue size and lymph node metastasis [17]. Thus, NOP14 may play an important role in the occurrence and development of melanoma tumors. Furthermore, NOP14 overexpression can significantly inhibit melanoma cell growth, migration, and invasion and can increase the level of apoptosis [17]. These findings suggest that NOP14 plays a role in regulating the pathogenesis of melanoma. Importantly, NOP14 overexpression can inhibit the Wnt/β-catenin signaling pathway [17], which is an important mechanism for regulating of CSC stemness [16]. To the best of our knowledge, this is the first report on the involvement of NOP14 in melanoma CSCs.

This study aimed to investigate the role and mechanism of NOP14 in the stemness and function of melanoma CSCs *in vitro*. We hypothesized that NOP14 might affect melanoma CSC stemness and function via the Wnt/β-catenin signaling pathway.

## Materials and methods

### Cell culture

The human melanoma cell line A375 was purchased from the Cell Bank of the Chinese Academy of Sciences (Shanghai, China). The A875 human melanoma cell line was purchased from China Center for Type Culture Collection (Wuhan, China). All cells were cultured as previously described [17].

### Construction of stable NOP14 Overexpression cells

PCR amplification of the full-length human NOP14 coding sequence (NM_001291978.2) was conducted using the following primer pairs: 5’-CGCGGATCCGCCACCATGGCG AAGGCGAAGAAGGTCGGGGCG-3’ and 5’-CCGACGCGTTTTTTTGAACTTTTTCCTCTTCAGA-3’. The PCR products and pLVX-C-FLAG-PGK-Puro vector were digested using *Mlu*I and *Bam*HI. Purified DNA fragments were ligated using T4 DNA ligase (New England Biolabs, Ipswich, MA, USA). The recombinant plasmid was named LVX-hNOP14. LVX-hNOP14 and the lentiviral packaging vectors were transfected into 293 T cells to produce NOP14 expressing lentiviruses (lv-NOP14). Empty lentiviruses were used as a control and named lv-mock. Concentrated lv-NOP14 and lv-control particle titers were measured. A375 and A875 cells were infected with lv-NOP14 or lv-control particles (multiplicity of infection = 50). The lentivirus particles were then added and left for 48 h. Puromycin (5 μg/ml) was then added to the culture medium for screening performed over two weeks. Finally, the mixed A375 and A875 clone cells overexpressing NOP14 were named ov-NOP14, and the mixed A375 and A875 clone cells infected with lv-control were named mock.

### Treatment using the Wnt signaling activator BML-284

BML-284 (purity: 99.90%) was purchased from MedChemExpress (Monmouth Junction, NJ, USA). A stock BML-284 solution (1 mM) was prepared according to the supplier’s instructions. BML-284 was used to treat the A375 and A875 cells of the ov-NOP14 group at a concentration of 10 µM. The ov-NOP14 cells were treated with dimethylsulfoxide (DMSO) and used as the control.

### Measurement of CD133-positive (CD133^+^) cells

The A375 and A875 cells of the mock and ov-NOP14 groups (1 × 10^6^) were harvested. APC-labeled anti-CD133 antibodies diluted in phosphate-buffered saline (PBS) were added to the cell suspension. The suspension was incubated in dark for 30 min, and the cells were washed twice with PBS. Finally, the cells were resuspended in PBS, and the percentage of CD133^+^ cells was measured using a BD FACSCalibur Flow Cytometer (BD Biosciences, San Diego, CA, USA) [18].

### Cell colony formation assay

A cell colony formation assay based on the reports of Vijayalakshmi Rajendran and Mayur Vilas Jain was performed to assess the CSC activity [19]. The A375 and A875 cells from different groups were seeded in 12-well culture plates (300 cells per well). The culture plate was shaken back and forth horizontally to ensure an even distribution. The culture medium was changed every two days, until day 14. We then aspirated the medium in each well, rinsed the wells twice with PBS, and added 500 µl of crystal violet staining solution to submerge the bottom of the well. After 20 min, the 12-well plate was rinsed using slow-flowing tap water. The specimens were dried, and a camera was used to photograph the whole well. Two experienced researchers manually counted the number of colonies containing >50 cells.

### Western blotting

The A375 and A875 cells from different groups were harvested, and total protein was isolated for western blotting. Western blotting was performed as previously described [17] to measure the protein levels of NOP14, Nanog homeobox (Nanog), SRY-box transcription factor 2 (SOX2), octamer-binding transcription factor 4 (OCT4), wingless-type MMTV integration site family member 3A (WNT3A), β-catenin, phosphorylated β-catenin (p-β-catenin, phosphorylation at Ser33, Ser37, and Thr41), and glycogen synthase kinase 3 beta (GSK-3β). Glyceraldehyde-3-phosphate dehydrogenase (GAPDH) was used as the loading control. The dilutions of the primary antibodies were as follows: anti-NOP14 (1:2000; ab157112), anti-Nanog (1:1000; #4903), anti-SOX2 (1:1000; #3579), anti-OCT4 (1:1500; # 2750), anti-WNT3A (1:1000; #2391), anti-p-β-catenin (1:1000; #9561), anti-β-catenin (1:1000; #8480), anti-GSK3β (1:1000; #9315), and anti-GAPDH (1:1000; #5174). The anti-NOP14 antibody was purchased from Abcam (Cambridge, MA, USA). Other primary antibodies were purchased from Cell Signaling Technology, Inc. (Danvers, MA, USA). Relative protein expression levels were measured as described by Guo and Xia [20].

### Sorting of Melanoma Stem-Like Cells (SLCs)

Anti-CD133 antibodies were used to sort the melanoma SLCs of the A375 and A875 cells. The sorted cells were named A375-SLC and A875-SLC and labeled using the Miltenyi Biotec CD133 MicroBead Kit (Miltenyi Biotec, Bergisch Gladbach, Germany) instructions. A375-SLCs and A875-SLCs were separated using a miniMACS Separator (Miltenyi Biotec). The sorted CD133^+^ A375-SLCs and CD133^+^ A875-SLCs were cultured and used for subsequent assays.

### Transwell migration and invasion assay

The migration and invasion capabilities of the A375-SLCs and A875-SLCs in different groups were assessed using the Transwell method as previously described [17].

### Cell proliferation measurements of human umbilical vein endothelial cells (HUVECs) using the transwell co-culture method

HUVECs (1 × 10^5^ cells) were inoculated in the lower chamber of the 24-well Transwell chamber. A375-SLCs and A875-SLCs (4 × 10^4^ cells) of each group were added to the upper chamber on the following day. The suspension was co-cultured for 24 h, and the cells and culture medium of the lower chamber were collected and seeded in 96-well culture plates. The absorbance was measured at 450 nm (OD _450 nm_) after 24, 48, and 72 h to assess the cell proliferation ability, according to the instructions of the Cell Counting Kit-8 (Dojindo) [21].

### HUVECs matrigel angiogenesis assay using the transwell co-culture method

The lower chamber of the 24-well Transwell chamber was coated with Matrigel one day before the experiment. HUVECs (1 × 10^5^ cells) were inoculated onto the Matrigel in the lower chamber. A375-SLCs and A875-SLCs (4 × 10^4^ cells) from each group were added to the upper chamber. Culturing was carried out for 6 h and pictures were taken using a 100-fold phase-contrast microscope. The branch nodes and tubes were counted under five fields, and the average number was used to assess angiogenesis [22].

### Luciferase-based T-cell factor (TCF) reporter assay

TOPflash plasmids (Wuhan Miaoling Biological Technology, China) with firefly luciferase were used as TCF reporters [23]. The pRL-TK plasmid (Promega) with *Renilla* luciferase was used as an internal control. A375 and A875 cells of the mock and ov-NOP14 groups (1 × 10^6^) were transiently co-transfected with TOPflash and pRL-TK. The cells were lysed with 5× passive lysis buffer (Promega) after 48 h, and the firefly and *Renilla* luciferase activity was measured using the Dual-Luciferase® Reporter Assay System (Promega) and a Fluoroskan Ascent FL microplate reader (Thermo Scientific, Waltham, MA, USA). The firefly luciferase activity was standardized against the *Renilla* luciferase activity. Relative luciferase activity was used to evaluate β-catenin-dependent signaling events.

### Statistical analysis

SPSS Statistics software for Windows (version 19.0; IBM Corp., Armonk, NY, USA) was used for statistical analysis. All statistical data from the independently repeated experiments were expressed as mean ± standard deviation. The statistical method used was the *t*-test. Differences were considered statistically significant at *P* < 0.05.

## Results

We hypothesized that NOP14 might affect melanoma CSCs. To test this, we analyzed the effect of NOP14 overexpression on CD133^+^ cell population levels, stem cell marker protein levels, and the colony-forming capabilities of A375 and A875 cells. We also investigated the effect of NOP14 overexpression on migration, invasion, and angiogenesis-inducing ability and explored the key proteins involved in Wnt/β-catenin signaling in A375-SLCs and A875-SLCs. The Wnt signaling activator BML-284 was used to further evaluate whether NOP14 functions through Wnt signaling.

### NOP14 overexpression suppressed melanoma cell stemness

**Figure 1.**
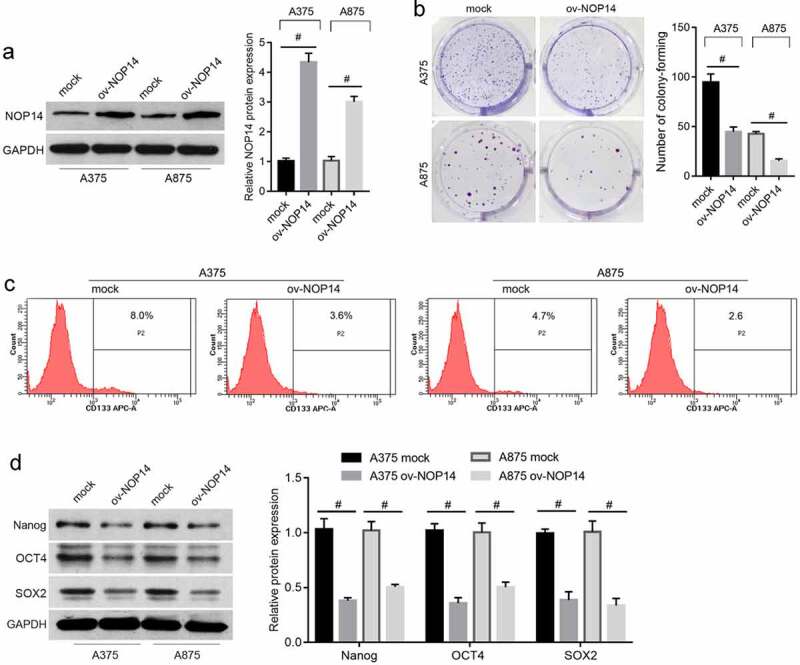
NOP14 overexpression suppressed melanoma cell stemness. A375 and A875 cells overexpressing NOP14 were grouped as ov-NOP14, and the control group was named mock. (a): NOP14 overexpression was verified using western blotting. B–D: NOP14 overexpression suppressed cell colony formation (b); decreased the CD133^+^ cell percentage; (c) and downregulated Nanog, SOX2, and OCT4 protein expression (d), as analyzed using cell colony formation assays, flow cytometry analysis, and western blotting. #*P* < 0.05 for the ov-NOP14 group versus the mock group.

The ov-NOP14 group had higher NOP14 protein levels than the mock group, indicating that NOP14 was successfully overexpressed in A375 and A875 cells (Figure 1(a)). The number of colony-forming units (Figure 1(b)) and percentage of CD133^+^ cells (Figure 1(c)) were lower in the ov-NOP14 group than in the mock group. NOP14 overexpression also decreased the Nanog, SOX2, and OCT4 stem cell marker protein levels (Figure 1(d)).

### NOP14 Overexpression Suppresses Melanoma CD133^+^ SLC Functioning

**Figure 2.**
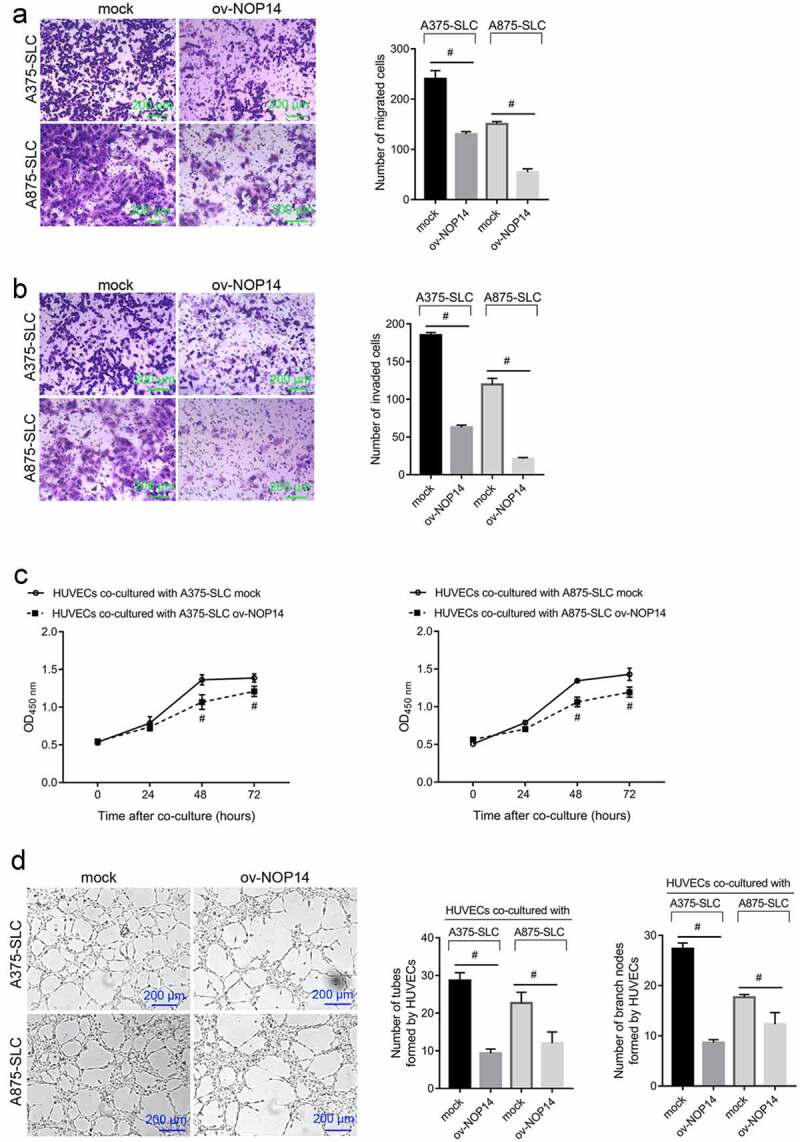
NOP14 overexpression suppressed the migration, invasion, and angiogenesis-induction ability of melanoma CD133^+^ SLCs. A375 and A875 cells overexpressing NOP14 were named ov-NOP14, and the control group was named mock. SLCs were sorted using anti-CD133 antibodies and named A375-SLC and A875-SLC. A–B: NOP14 overexpression suppressed the (a) migration and (b) invasion capabilities of A375-SLCs and A875-SLCs. (c–d): A375-SLCs and A875-SLCs overexpressing NOP14 suppressed (c) the cell proliferation and (d) tube-forming capabilities of HUVECs. ^#^*P* < 0.05 for the ov-NOP14 group versus the mock group.

We sorted CD133^+^ A375-SLCs and A875-SLCs and assessed how NOP14 overexpression affected their migration and invasion. The number of migrated and invaded A375-SLCs and A875-SLCs was lower in the ov-NOP14 group than in the mock group, suggesting that NOP14 overexpression reduced their migration and invasion capabilities (Figure 2(a,b)). Furthermore, A375-SLCs and A875-SLCs were co-cultured with HUVECs to analyze the effect of NOP14 on CSC-induced angiogenesis. The OD_450 nm_ value of HUVECs co-cultured with A375-SLC ov-NOP14 was lower than that of the HUVECs co-cultured with A375-SLC mock (Figure 2(c)). Moreover, the number of tubes and branch nodes formed by HUVECs co-cultured with A375-SLC ov-NOP14 were lower than that of HUVECs co-cultured with A375-SLC mock (Figure 2(d)). A875-SLCs overexpressing NOP14 had similar effects on HUVECs (Figure 2(c,d)). NOP14 overexpression also reduced A375-SLC-SLC- and A875-SLC-induced angiogenesis (Figure 2(c,d)).

### NOP14 overexpression inactivated Wnt/β-catenin signaling in melanoma CD133^+^ SLCs

**Figure 3.**
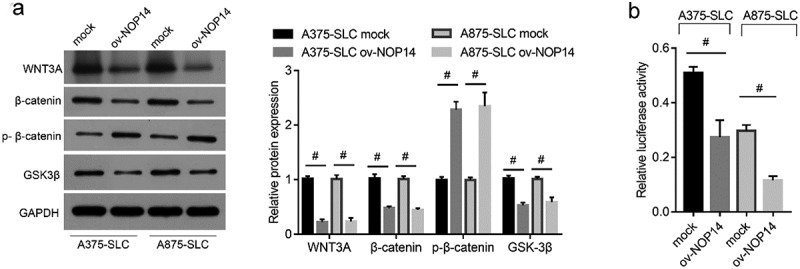
NOP14 overexpression inactivated Wnt/β-catenin signaling in melanoma CD133^+^ SLCs. A375 and A875 cells overexpressing NOP14 were named ov-NOP14, and the control group was named mock. The SLCs of each group were sorted using anti-CD133 antibodies and named A375-SLC and A875-SLC. (a): The WNT3A, β-catenin, phosphorylated β-catenin (p-β-catenin), and GSK-3β protein levels were assessed using western blotting. (b): The relative luciferase activity of the TCF reporter in the mock and ov-NOP14 groups. ^#^*P* < 0.05 for the ov-NOP14 group versus the mock group.

Wnt/β-catenin is a key signaling regulator in CSCs. Therefore, we investigated the effect of NOP14 overexpression on the key proteins involved in Wnt/β-catenin signaling in CD133^+^ A375-SLCs and A875-SLCs. The WNT3A, β-catenin, and GSK-3β protein levels were lower, and the p-β-catenin (phosphorylation at Ser33, Ser37, and Thr41) protein levels were higher, in the ov-NOP14 group (Figure 3(a)). β-catenin induces the transcriptional activation of TCF. Therefore, a luciferase-based TCF reporter assay was carried out to assess β-catenin activity. The relative luciferase activity of the TCF reporter was lower in the ov-NOP14 group (Figure 3(b)), indicating that NOP14 overexpression suppresses β-catenin activity.

### BML-284 Alleviated the Effect of NOP14 Overexpression on Melanoma Cell Stemness

**Figure 4.**
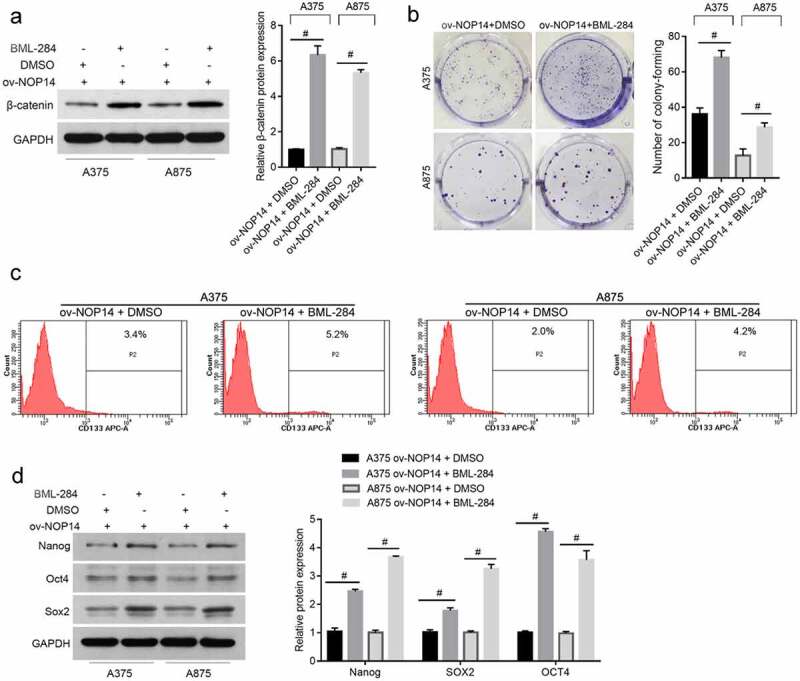
The Wnt signaling activator, BML-284, alleviated the effect of NOP14 overexpression on the stemness of melanoma cells. The A375 and A875 cells of the ov-NOP14 group were treated with BML-284 or DMSO. (a): β-catenin expression was verified using western blotting. (b–d): BML-284 treatment promoted cell colony formation (b), increased the percentage of CD133^+^ cells (c), and upregulated Nanog, SOX2, and OCT4 protein expression (d), as analyzed using cell colony formation assays, flow cytometry, and western blotting, respectively. #*P* < 0.05 for the ov-NOP14 + BML-284 group versus the ov-NOP14 + DMSO group.

Whether NOP14 affects SLCs through Wnt/β-catenin signaling was confirmed using the Wnt signaling activator BML-284 to rescue the effect of NOP14 overexpression on Wnt/β-catenin signaling. β-catenin expression was successfully upregulated in the ov-NOP14 group (Figure 4(a)). The number of colony-forming units and percentage of CD133^+^ cells was higher in the ov-NOP14 + BML-284 group than in the ov-NOP14 + DMSO group (Figure 4(b,c)). Moreover, the Nanog, SOX2, and OCT4 protein levels were higher in the ov-NOP14 + BML-284 group than in the ov-NOP14 + DMSO group (Figure 4(d)). These results indicated that BML-284 alleviated the effect of NOP14 overexpression on the stemness of melanoma cells.

### BML-284 Alleviated the Effect of NOP14 Overexpression on the Function of Melanoma CD133^+^ SLCs

**Figure 5.**
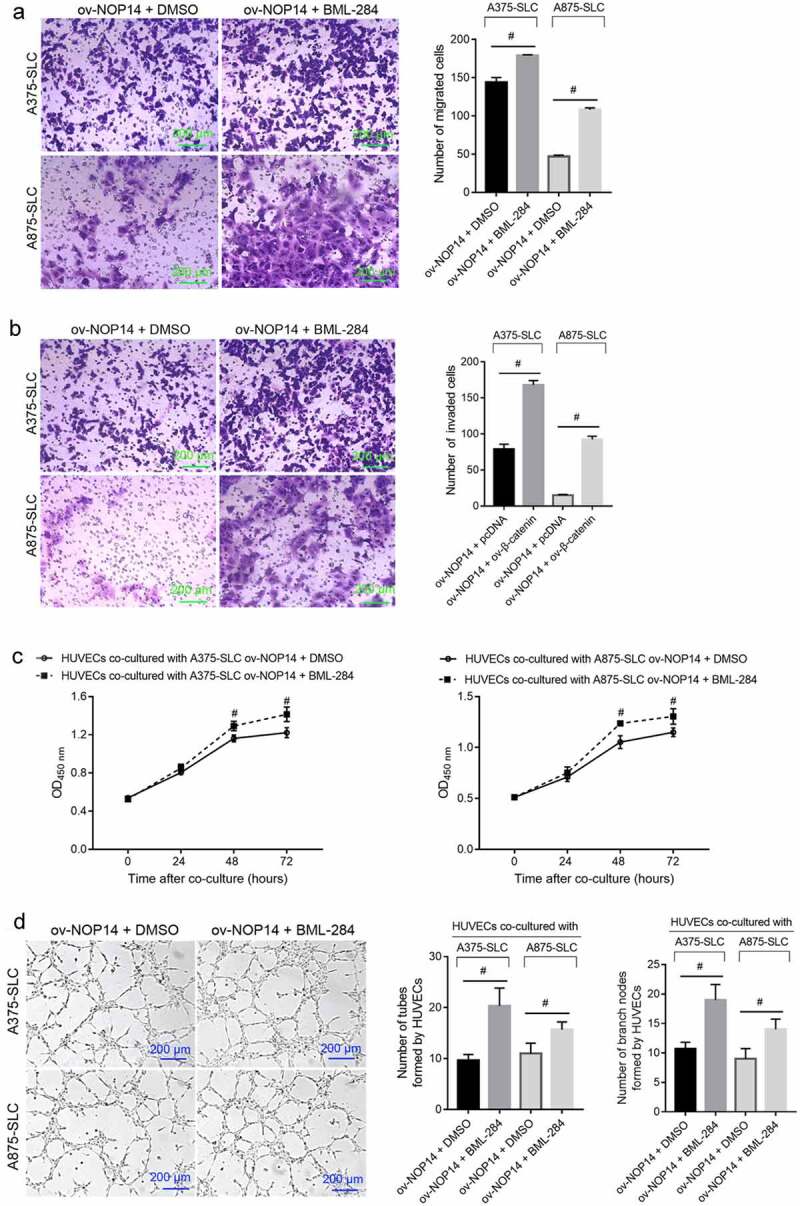
The Wnt signaling activator BML-284 alleviated the suppressive effect of NOP14 overexpression on the migration, invasion, and angiogenesis-inducing ability of melanoma CD133^+^ SLCs. After treating the A375 and A875 cells of the ov-NOP14 group with BML-284 or DMSO, CD133^+^ A375-SLC and A875-SLC were sorted. (a–b): BML-284 treatment the (a) migration and (b) invasion capabilities of A375-SLCs and A875-SLCs. C–D: BML-284 treatment promoted the (c) cell proliferation and (d) tube-forming capabilities of A375-SLC and A875-SLC HUVECs.

The number of migrated and invaded CD133^+^ A375-SLCs and A875-SLCs was higher in the ov-NOP14 + BML-284 group than in the ov-NOP14 + DMSO group, suggesting that BML-284 alleviated the suppressive effect of NOP14 overexpression (Figure 5(a,b)). Furthermore, the A375-SLCs and A875-SLCs of the ov-NOP14 + BML-284 and ov-NOP14 + DMSO groups were co-cultured with HUVECs to analyze their effect on angiogenesis. The OD_450_ nm value of HUVECs co-cultured with the A375-SLCs of the ov-NOP14 + BML-284 group was higher than that of HUVECs co-cultured with the ov-NOP14 + DMSO group (Figure 5(c)). Moreover, the number of tubes and branch nodes formed by HUVECs co-cultured with the A375-SLCs of the ov-NOP14 + BML-284 group was greater than that of HUVECs co-cultured with the A375-SLCs of the ov-NOP14 + DMSO group (Figure 5(d)). The A875-SLCs of each group had a similar effect on HUVECs (Figure 5(c,d)). Thus, BML-284 alleviated the suppressive effect of NOP14 overexpression on the ability of A375-SLCs to induce angiogenesis (Figure 5(c,d)).

## Discussion

This study elucidated the critical role and underlying mechanisms of NOP14 in melanoma CSC development. NOP14 overexpression suppressed melanoma cell stemness, reduced migration, invasion, and angiogenesis-inducing capabilities of melanoma CD133^+^ SLCs. NOP14 overexpression inactivated Wnt/β-catenin signaling in melanoma CD133^+^ SLCs. Moreover, the Wnt signaling activator BML-284 alleviated the effect of NOP14 overexpression.

CD133, also known as prominin-1, is a pentaspan membrane glycoprotein widely used to isolate and identify CSCs [24]. Only CD133 was used to isolate melanoma CSCs in this study. The design of this study is supported by various publications, including a report by He et al. [25]. NOP14 overexpression decreased the proportion of CD133^+^ SLCs in A375 and A875 cells. This suggested that NOP14 overexpression reduced the proportion of melanoma CSCs. In addition, NOP14 overexpression inhibited the colony-forming capabilities and downregulated the expression of Nanog, SOX2, and OCT4 stem cell markers in A375 and A875 cells. CSCs have stronger clonogenic capabilities and higher stem cell marker expression levels than cancer cells without stemness. Differences in the clonogenic ability and stem cell marker expression were assessed in whole melanoma cell populations. These changes may be caused by proliferation suppression and stemness reduction of melanoma CSCs. The effect of NOP14 on the proportion of CD133^+^ SLCs was minor. Thus, we speculate that NOP14 may function through a suppressive effect on stemness in melanoma CSCs.

Cancer recurrence and metastasis are closely related to CSCs [9], which have significant potential to metastasize [9]. Inhibiting the migration and invasion of CSCs can fundamentally inhibit cancer recurrence and metastasis. NOP14 overexpression suppressed the migration and invasion of melanoma CD133^+^ SLCs. Therefore, we hypothesized that NOP14 could function as a metastasis suppressor in melanoma CSCs. A close relationship between CSCs and angiogenesis was previously suggested [26,27]. CSCs may differentiate into endothelial cells to participate in angiogenesis or the regulation of angiogenesis by secreting pro-angiogenic, matrix-derived, and hypoxia-inducible factors. This study investigated the effect of CD133^+^ SLCs on HUVECs through non-contact co-culturing. NOP14 overexpression impaired the induction of angiogenesis in melanoma CD133^+^ SLCs. Therefore, NOP14 can affect CD133^+^ SLC secretion, which will be further investigated in future studies. In summary, our results demonstrated that NOP14 overexpression affects the stemness and functions of melanoma CSCs.

Our results showed that NOP14 decreased WNT3A, β-catenin, and GSK-3β protein levels but increased the p-β-catenin (phosphorylation at Ser33, Ser37, and Thr41) protein levels. WNT3A, β-catenin, and GSK-3β are key Wnt signaling proteins [28]. Phosphorylating β-catenin at Ser33, Ser37, and Thr41 destabilizes β-catenin and results in β-catenin degradation [29]. Thus, NOP14 overexpression inactivated Wnt/β-catenin signaling in melanoma CD133^+^ SLCs. This is further supported by the suppressive effect of NOP14 on β-catenin activity, measured using a luciferase-based TCF reporter assay. Wnt/β-catenin signaling regulates stem cell regeneration in normal somatic stem cells and CSCs [30]. The Wnt signaling activator BML-284 also alleviated the effect of NOP14 overexpression on the stemness and function of melanoma cells. Therefore, our results support the notion that NOP14 suppresses the stemness and function of melanoma SLCs by inactivating Wnt/β-catenin signaling.

This study has some limitations. First, the effect of NOP14 on stemness in sorted CD133^+^ cells and their tumor-forming ability was not confirmed using animal models. Second, the effect of NOP14 on other signaling pathways involved in stemness regulation was not investigated. Further investigations on the function and molecular mechanism of NOP14 should be conducted.

## Conclusion

In conclusion, our results showed that NOP14 suppresses the stemness and function of melanoma SLCs by inactivating Wnt/β-catenin signaling. Our study identified a novel target for melanoma CSC therapy. We plan to verify this conclusion using animal models in future investigations to further refine our knowledge about the regulatory mechanisms.

## Data Availability

All data from this study are available in this published article.
